# Effectiveness of a primary care-based intervention to reduce sitting time in overweight and obese patients (SEDESTACTIV): a randomized controlled trial; rationale and study design

**DOI:** 10.1186/1471-2458-14-228

**Published:** 2014-03-05

**Authors:** Carme Martín-Borràs, Maria Giné-Garriga, Elena Martínez, Carlos Martín-Cantera, Elisa Puigdoménech, Mercè Solà, Eva Castillo, Angela Mª Beltrán, Anna Puig-Ribera, José Manuel Trujillo, Olga Pueyo, Javier Pueyo, Beatriz Rodríguez, Noemí Serra-Paya

**Affiliations:** 1 Research Unit of Barcelona, Primary Healthcare Research Institution IDIAP Jordi Gol, Barcelona, Spain; 2 Department of Physical Activity and Sport Sciences, FPCEE Blanquerna, Universitat Ramon Llull, Barcelona, Spain; 3 Department of Physical Therapy, FCS Blanquerna, Universitat Ramon Llull, Barcelona, Spain; 4 Primary heatlhcare centre Vilanova, Institut Català de la Salut, Barcelona, Spain; 5 Primary healthcare centre Passeig Sant Joan, Institut Català de la Salut, Barcelona, Spain; 6 Department of Medicine, Universitat Autònoma de Barcelona, Barcelona, Spain; 7 Primary healthcare centre Les Planes, Institut Català de la Salut, Barcelona, Spain; 8 Primary healthcare centre Sant Ildefons Cornellà, Institut Català de la Salut, Cerdanyola-Ripollet, Spain; 9 Department of Physical Activity Sciences, Universitat de Vic, Vic, Spain; 10 Primary healthcare centre Cuevas del Almanzora, Servicio Andaluz de Salud, Almería, Spain; 11 Primary healthcare centre Cariñena, Servicio Aragonés de Salud, Zaragoza, Spain; 12 Fremap, Zaragoza, Spain; 13 National Institute for Physical Education of Catalonia (INEFC) of Lleida, Universitat de Lleida, Lleida, Spain; 14 SEDESTACTIV Study Group, redIAPP: Red de Investigación en Actividades Preventivas y Promoción de la Salud, Barcelona, Spain

**Keywords:** Sedentary behaviour, Sitting time, Primary care, Overweight, Obese patients

## Abstract

**Background:**

There is growing evidence suggesting that prolonged sitting has negative effects on people’s weight, chronic diseases and mortality. Interventions to reduce sedentary time can be an effective strategy to increase daily energy expenditure. The purpose of this study is to evaluate the effectiveness of a six-month primary care intervention to reduce daily of sitting time in overweight and mild obese sedentary patients.

**Method/Design:**

The study is a randomized controlled trial (RCT). Professionals from thirteen primary health care centers (PHC) will randomly invite to participate mild obese or overweight patients of both gender, aged between 25 and 65 years old, who spend 6 hours at least daily sitting. A total of 232 subjects will be randomly allocated to an intervention (IG) and control group (CG) (116 individuals each group). In addition, 50 subjects with fibromyalgia will be included.

Primary outcome is: (1) sitting time using the activPAL device and the Marshall questionnaire. The following parameters will be also assessed: (2) sitting time in work place (Occupational Sitting and Physical Activity Questionnaire), (3) health-related quality of life (EQ-5D), (4) evolution of stage of change (Prochaska and DiClemente's Stages of Change Model), (5) physical inactivity (catalan version of Brief Physical Activity Assessment Tool), (6) number of steps walked (pedometer and activPAL), (7) control based on analysis (triglycerides, total cholesterol, HDL, LDL, glycemia and, glycated haemoglobin in diabetic patients) and (8) blood pressure and anthropometric variables. All parameters will be assessed pre and post intervention and there will be a follow up three, six and twelve months after the intervention. A descriptive analysis of all variables and a multivariate analysis to assess differences among groups will be undertaken. Multivariate analysis will be carried out to assess time changes of dependent variables. All the analysis will be done under the intention to treat principle.

**Discussion:**

If the SEDESTACTIV intervention shows its effectiveness in reducing sitting time, health professionals would have a low-cost intervention tool for sedentary overweight and obese patients management.

**Trial registration:**

A service of the U.S. National Institutes of Health. Developed by the National Library of Medicine. ClinicalTrials.gov NCT01729936

## Background

Obesity is one of the main leading cause of mortality worldwide; in fact according to the World Health Organization (WHO) more than 2.8 million adults die each year because they are obese or overweight. In addition, is one of the main risk factor for many of the leading causes of mortality such as cardiovascular diseases, stroke and certain cancers [1]. The percentage of obese and overweight people worldwide has significantly risen in the past years [2]. Therefore, prevention and reduction of obesity should be a public health priority and mainly include: rising physical activity (PA), lowering sedentary behavior (SB) and modifying the diet to low-calorie and low-fat diets [3].

Sedentary behavior is any waking behavior characterized by an energy expenditure ≤1.5 Metabolic Equivalent Units (METs) while in a sitting or reclining posture [4]. Recently, a range of observational data has reported associations between sedentary time and adverse health outcomes [5], including an increased risk of diabetes, cardiovascular and all-cause mortality [6-8].

Lifestyle changes in high-income countries have led to a decrease in the energy expenditure needed for daily life, an increase in sedentary activities and weight gain. Levels of PA and sedentary behavior among young people tend to promote obesity [9].

The dose–response relationship among amount of PA, energy expenditure and weight loss is obvious [10]. People with overweight and obesity should accomplish 5 sessions of 45–60 minutes of mild-intensity PA per week [11]. Unfortunately, evidence shows the difficulty of overweight and obese population to achieve these recommendations in the long term; PA dropout among them is quite high [12].

In this context, and considering the scarce adherence to PA in people with overweight and obesity, there is recent evidence that interventions to increase energy expenditure perhaps should first focus on reducing time of sedentary activities. It is well known that walking is a simple PA accessible to most people, which can have an important role to increase daily energy expenditure and help reduce SB [13]. Moreover, if obese people could manage to be standing or wandering for 2.5 hours per day instead of sitting, this would represent an extra energy expenditure of 350 kcal per day, and they might find it easier to maintain this activity in the long term [14].

To effectively manage the increasing number of patients with obesity-related chronic illnesses, the medical community should embrace a major role in encouraging patients to make healthy lifestyle choices. Primary care is well suited to fill this need, because most of the population identifies a primary care clinician as their usual source of care [15].

There are few randomized controlled trials that assess the impact of interventions on the reduction in daily sitting time. Additionally, these studies have included small samples. A recent intervention with 92 subjects with type-2 diabetes achieved a reduction in daily sitting time of 23 min/day [16]. Another study with 12 patients with overweight and obesity showed an increase in walking time per day (146 to 203 min/day), increase in number of steps/day (4351 to 7080 steps/day) and decrease in sitting time (1238 to 1150 min/day) [17]. To our knowledge, there is no evidence on the effectiveness of an intervention based on reducing daily sitting time in adults with overweight and mild obesity and its sustainability in the long term.

The aim of the present study is to assess whether a sustained reduction of sedentary time is possible, and if so, to evaluate the effectiveness of an education-based intervention among overweight and mild obese adults attended in Primary Care in terms of reduction of sitting time. Secondary aims are to determine health benefits of the intervention among studied population.

## Method/Design

The present study is a randomized controlled trial (RCT). The intervention evaluated is education-based in order to reduce sedestation time among overweight and mild obese adults attending primary care. The Project SEDESTACTIV includes two phases. Phase 1: a qualitative study already undertaken to know the proposals, barriers and facilitators of overweight or mild obesity users of primary health care (PHC) on reducing sitting time. Phase 2: a randomized controlled intervention trial based on education to reduce sedentary behavior (SB).

### Phase 1. Qualitative data collection and analysis

The qualitative study included focal groups and semi-structured interviews were conducted by an expert moderator and an observer in PHC. 22 overweight or mild obese aged 25–65 years who were 6 or more hours sitting (see study population) participated. Awareness, acceptability and readiness to reduce sedentary behavior (according to importance, motivation and confidence) and opinions about primary care interventions to reduce sitting time were discussed. Facilitators and barriers for change were also asked. The number of subjects was determined by the saturation of the information (n = 22). All participants read and signed the informed consent form. All interviews were recorded, transcribed and analyzed using Atlas.ti, performing a triangulation of analysis. Ethical approval was granted by Clinical Investigation Ethics Committee of the IDIAP Jordi Gol, located in Barcelona.

Qualitative data was used to design the RCT education intervention to reduce sitting time. From these findings we can say that the interventions from PHC to reduce SB in overweight and mild obesity people should include monitoring by a professional and face-to-face follow-up.

### Phase 2. Randomized controlled trial (RCT)

Phase 2 consist of a multi-centric RCT to assess the effectiveness of a six-month primary care intervention to reduce the daily hours of sedestation as well as to increase their weekly caloric spend at the end of the intervention and at short, medium and long term among overweight and mild obese persons. The CONSORT (Consolidated Standards of Reporting Trials) guidelines for RCT were used to design the study and will be used to conduct it [18].

### Study population

Inclusion criteria of participants will include: (a) men and women aged 25–65 seen at the PHC for whatever reason; (b) diagnosed of being overweight or suffer mild obesity (BMI: 25–34.9 kg/m^2^); (c) autonomous subjects who have minimum physical aptitudes to follow the recommendations (being able to walk and stand up from a chair independently); (d) who are ≥6 hours daily sitting; and (e) who can assure its participation in the study for a year.

Exclusion criteria will be based on certain medical conditions which could contraindicate the fulfillment of the intervention. Patients who have had obesity surgery will be also excluded.

Written informed consent will be obtained from all subjects of both intervention (IG) and control group (CG) prior to its inclusion on the study. This trial was approved by the Clinical Investigation Ethics Committee of the IDIAP Jordi Gol and registered at trial NCT01729936.

### Recruitment process and randomisation

**Figure 1:**
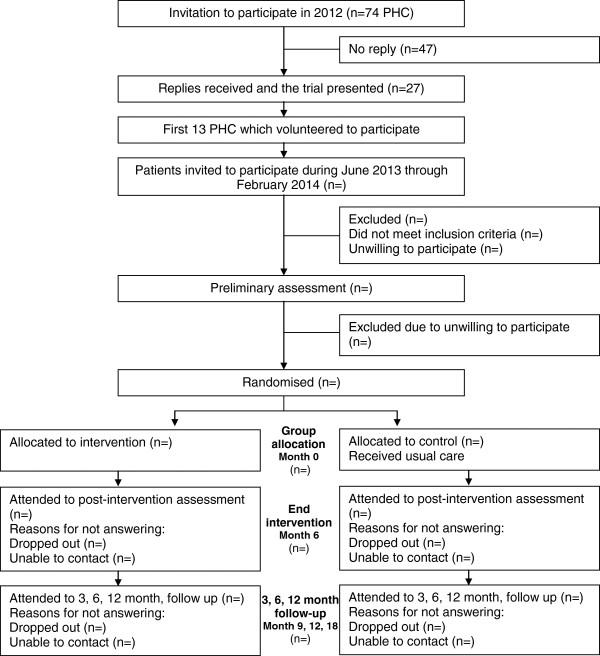
Flowchart of participant’s recruitment and trial design.

Recruitment will take place in 13 PHC in different regions of Spain (Barcelona, Lleida, Zaragoza and Almeria). Primary health care professionals, who were selected on a voluntary basis from each of the participating centers, were trained. During the recruitment period, the opportunity to participate in the study will be offered at least once a week to all patients with overweight or mild obesity. A total of 30 subjects will be recruited in each PHC. Patients who meet the inclusion criteria and agree to participate will be contacted and informed about how the project will be carried out. All of them will be requested to sign the informed consent form to take part in the study. Demographic and health data will be recorded for all participants.Random sequence generation will be computer-generated by an independent researcher, guaranteeing that the allocation groups remain confidential. Participants will be randomized to intervention group (IG) or control group (CG) after baseline measures assessment. Figure 1 shows the flowchart of participants’ recruitment and trial design.

### Description of the intervention

#### Intervention group

The intervention will be done according to the patient’s willingness to reduce sitting time (stage of change), and considering whether the subject prefers a more or less intensive intervention: (a) precontemplative (not thinking about change) and contemplative (thinking about change), (b) ready to change with minimum professional support, or (c) ready to change with intensive professional support. All of them will be handed a card with the planned sessions and Mediterranean diet recommendations.

Precontemplative and contemplative patients (a) will be informed of the risks of being 6 or more hours sitting and will be asked about the importance and confidence for change. Ready to change patients (b and c) will be proposed an intervention based on 2–5 meetings (visits face-to-face or phone calls depending on the situation of each professional and patient) with a trained professional during 6 months. On those meetings, the professional will work on finding alternatives to progressively reduce SB by developing the same activities walking.

### Control group

Subjects assigned to the control group will be given information on the study and will be asked to continue their routine daily activities. They will receive their usual care from their primary care practice. Furthermore, the health professional will give the patient pamphlet with the Mediterranean diet recommendations. They will be followed at the end of intervention and at three, six and twelve months follow-up. At the following visits several parameters will be evaluated (see Outcome measures).

### Blinding – single blind

Baseline measures will be taken prior to allocation of randomisation. Independent investigators assessing participants at the end of the intervention and at three, six and 12 month follow-up visits or phone calls will be blind to the allocation of the treatment group. Participants will be asked not to discuss group allocation with the assessing professional. Moreover, the person who carries out the analysis of the data will not be involved in the investigation.

### Outcome measures

#### Main objective

The primary outcome is sitting time measured by using the activPAL device (PAL technologies, UK). It also will be measured with the Marshall questionnaire [19]. The study measure will be assessed at baseline, at the end of the intervention, and at three, six and twelve months follow-up.

Sitting time will be evaluated, moving from ≥6 hours daily sitting to less sitting time, and increasing the weekly number of METs. The activPAL enables to evaluate caloric expenditure, time sitting/lying, number of steps, standing time, walking time and the transitions from one position to another. This instrument has shown good validity and reliability in general population in a previous study [20]. Marshall questionnaire enables sitting time to be evaluated as a continuous variable by calculating the weekly minutes sitting, and classifying the subjects according to whether they are more or less hours sitting. This instrument has shown good validity and reliability in general population in a previous study [19].

#### Other specific objectives

Secondary outcomes for IG and CG include: (a) decreasing sitting time in work place in individuals involved in the intervention, measured with Occupational Sitting and Physical Activity Questionnaire (OSPAQ) [21]; (b) description of the development of health-related quality of life measured with EQ-5D (Euroqool Group: http://www.euroqol.org) (first and last session of the intervention, with follow up at three, six and twelve months after the intervention); (c) evaluation the evolution of the attitude towards the change in behaviours in relation to reduce sitting time using the Prochaska scale (Prochaska and DiClemente’s Stages of Change Model) [22]; (d) evaluation of physical inactivity, assessed with catalan version of Brief Physical Activity Assessment Tool (CBPAAT). The CBAFC enables physical inactivity as a categoric outcome and classifies subjects as ‘active enough’ or ‘insufficiently active’. This instrument showed good validity and reliability in general population in a previous study [23]; (e) evaluation of the number of daily steps walked, measured with DigiWalker 200w pedometer and activPAL posture monitor. Reliability and validity of these devices were previously assessed [20,24]; (f) evolution of anthropometric variables: BMI, triceps skinfold and waist circumference; and (g) description of the evolution of triglycerides, total cholesterol, HDL, LDL, glycemia and, glycated haemoglobin in diabetic patients (first and last session and 6 and 12 months after the intervention).

All parameters (not (g) outcome) will be assessed pre and post intervention in both groups and there will be a follow up in a face to face interview three, six and twelve months after the intervention.

Sociodemographic and health data will be also collected (age, marital status, educational level, blood pressure, BMI and associated pathologies).

### Data management and quality assurance

Data will be entered directly into an online questionnaire (SurveyMonkey: https://es.surveymonkey.com) by investigators at the time of the interview and baseline testing. Daily backups will be performed and transferred to the master database at least once a week. Random checks of data entry will be performed regularly and corrections made will be possible by checking against electronic records and by phoning professionals for patient’s information confirmation by independent investigators.

### Sample size

Since no previous intervention studies on caloric spend had been undertaken –similar studies report sedestation time- we consider that the current intervention is analogous to others proposed in other lifestyles such as tobacco consumption. Accepting an alpha risk of 0.05 and a beta risk < 0.10 in a bilateral contrast, 232 individuals are needed: 116 individuals in the IG and 116 in the CG in order to detect a difference ≥ 0.15 between the two. A proportion of 0.5 in one of the groups is to be assumed. A dropout rate of 20% is estimated. The sample size calculations were performed with the Granmo program (version 7.1).

### Statistical methods

Analysis of effectiveness will be made followed by the intention to treat analysis. Descriptive statistics will be computed as customary [25,26]. Control and intervention groups will be checked for health and outcome measures and a baseline comparability analysis of the CG and IG in relation to the variables studied, will be carried out. An t-Student test or ANOVA will be used in the comparison of means if the variables follow a normal distribution and the U of Mann Whitney if they do not. For the other dimensions of the analysis, a covariance analysis (ANCOVA) for repeated measures will be carried out.

Multi-variate analysis will evaluate the change in the time of the described dependent variables, and comparisons between the CG and IG will be established. To do this multilevel linear models will be adjusted, one for each dependent variable. In the first level the individual path or the evolution of each individual over a long period of time (pre-post intervention, 3, 6 and 12 month follow- up) will be modelled. In the second level it will be adjusted according to the variables that refer to the individual; the intervention variable will be added (intervention or control) to the independent variables described earlier.

The level of statistical significance will be set at 0.05, and all tests will be two-tailed. Statistical analyses will be conducted using SPSS, version 17.0 (SPSS Inc, Chicago, IL).

### Ethical approval

The study will be carried out according to the principles of the “Declaration of Helsinki” and subsequent revisions [27]. This study was reviewed and approved by the Clinical Investigation Ethics Committee of the IDIAP Jordi Gol, located in Barcelona. The participation of the subjects is strictly voluntary and withdrawal will not have any consequence on the management of their illness which will be carried out by their doctor strictly following the accepted international norms. The data will be treated with utmost confidentiality according to the Organic Law which regulates the confidentiality of computerized data (Protection of personal information Law 15/1999), and will be used exclusively for the purposes of this scientific investigation.

## Discussion

Sedentary behaviour (SB) in general and sitting time in particular is increasingly recognised as a health risk behaviour. Evidence suggests that SB is associated with increased cardiovascular disease, diabetes, obesity and mortality independent from physical activity (PA) level [6,7]. Overweight and obese people are more sedentary (they spend more hours sitting each day). In addition, they have shown poor rates of change and adherence to PA [28,29]. Thus, prevention and reduction of obesity should be a public health priority, and reducing SB could be an appropriate strategy [3].

Primary care centers (PHC) are an ideal setting to identify adults who spend extended periods of daily sitting, and to treat people with overweight and mild obesity. Furthermore, are a key scene to initiate a cost-effective SB intervention. PHC practitioners can play an important role in population health throughout lifestyle promotion [30]. A high percentage of the population visit their primary care professional at least once a year [31] and tend to have confidence with them. Research has shown that PHC practitioners’ suggestions have a high impact in patients’ everyday life [32].

Encouraging overweight and mild obese patients to reduce sitting time could be the first step to increase their daily caloric expenditure. There are few randomized controlled trials (RCT) that had assessed the impact of interventions on the reduction in daily sitting time. Additionally, these studies have included small samples. RCT to reduce SB in sedentary patients based on objective measures to assess daily sitting time are needed.

SEDESTACTIV is a RCT based on a PHC intervention carried out by a healthcare practitioner to assess whether a sustained reduction of sitting time among overweight and mild obese adults is possible, and if so, to evaluate the effectiveness in determining health benefits among the studied population. The design of the SEDESTACTIV study takes advantage of the direct contact and accessibility of PHC with patients.Figure 1 shows the consolidated standards of reporting trials (CONSORT) flow diagram.

## Abbreviations

WHO: World Health Organization; PA: Physical activity; SB: Sedentary behavior; METs: Metabolic Equivalent Units; RCT: Randomized controlled trial; PHC: Primary health care; IDIAP Jordi Gol: Primary Care Research Institute Jordi Gol; CONSORT: Consolidated Standards of Reporting Trials; BMI: Body mass index; IG: Intervention group; CG: Control group; OSPAQ: Occupational Sitting & Physical Activity Questionnaire; CBPAAT: Catalan version of Brief Physical Activity Assessment Tool; HDL: High-density lipoprotein; LDL: Low-density lipoprotein.

## Competing interest

The study authors declare that they have no competing interest.

## Authors’ contributions

CMB, MGG, EM, CMC, EP, MS, EC and AP were responsible for the conception and design of the study, CMB, MGG, EM, CMC, EP, MS, EC, MB, AP, OP, JP, BR, JMT and NS conceived and participated in the design of the questionnaires, as well as to the development of the study. CMB, MGG and EP wrote the firsts drafts and final version of the study protocol. All authors have performed a critical revision of this manuscript and the final version. The project will be developed by SEDESTACTIV Study Group.

## Pre-publication history

The pre-publication history for this paper can be accessed here:

http://www.biomedcentral.com/1471-2458/14/228/prepub

## References

[B1] World Health Organization Obesity and overweight fact sheet 2013 http://www.who.int/mediacentre/factsheets/fs311/en/

[B2] Smyth S Heron A Diabetes and obesity: the twin epidemics Nat Med 2006 12 Suppl 1 75 80 16397575 10.1038/nm0106-75

[B3] Expert panel on integrated guidelines for cardiovascular health and risk reduction in children and adolescents 2012 http://www.nhlbi.nih.gov/guidelines/cvd_ped/peds_guidelines_full.pdf 10.1542/peds.2009-2107C PMC4536582 22084329

[B4] Network SBR Letter to the editor: standardized use of the terms “sedentary” and “sedentary behaviours” Appl Physiol Nutr Metab 2012 37 540 542 10.1139/h2012-024 22540258

[B5] Hamilton MT Hamilton DG Zderic TW Role of low energy expenditure and sitting in obesity, metabolic syndrome, Type 2 diabetes, and cardiovascular disease Diabetes 2007 56 2655 2667 10.2337/db07-0882 17827399

[B6] Dunstan DW Salmon J Owen N Armstrong T Zimmet PZ Welborn TA Cameron AJ Dwyer T Jolley D Shaw JE Physical activity and television viewing in relation to risk of undiagnosed abnormal glucose metabolism in adults Diab Care 2004 27 2603 2609 10.2337/diacare.27.11.2603 15504993

[B7] Dunstan DW Salmon J Healy GN Shaw JE Jolley D Zimmet PZ Owen N Association of television viewing with fasting and 2-h postchallenge plasma glucose levels in adults without diagnosed diabetes Diabetes Care 2007 30 516 522 10.2337/dc06-1996 17327314

[B8] Katzmarzyk PT Church TS Craig CL Bouchard C Sitting time and mortality from all causes, cardiovascular disease, and cancer Med Sci Sport Exer 2009 41 Suppl 5 998 1005 10.1249/MSS.0b013e3181930355 19346988

[B9] Reilly JJ Tackling the obesity epidemic: new approaches Arch Dis Child 2006 91 724 726 10.1136/adc.2006.098855 16923855 PMC2082930

[B10] Donnelly JE Blair SN Jakicic JM Manore MM Rankin JW Smith BK American college of sports medicine position stand: appropriate physical activity intervention strategies for weight loss and prevention of weight regain for adults Med Sci Sports Exerc 2009 41 459 471 19127177 10.1249/MSS.0b013e3181949333

[B11] Scottish Intercollegiate Guidelines Network Management of obesity. A national clinical guideline 2010 http://www.sign.ac.uk/pdf/sign115.pdf

[B12] Stevens J Truesdale KP McClain JE Cai J The definition of weight maintenance Int J Obes 2006 30 391 399 10.1038/sj.ijo.0803175 16302013

[B13] Lee IM Buchner DM The importance of walking to public health Med Sci Sport Ex 2008 40 512 518 10.1249/MSS.0b013e31817c65d0 18562968

[B14] Levine JA Vander Weg MW Hill JO Non-exercise activity thermogenesis. The crounching tiger hidden dragon of societal weight gain Artherior Thromb Vasc Biol 2006 26 729 736 10.1161/01.ATV.0000205848.83210.73 16439708

[B15] Fryer GE Analysis of Medical Expenditure Panel Survey (MEPS). The importance of having a usual source of health care Am Fam Physician 2000 62 Suppl 3 477 18853527

[B16] De Greef KP Deforche BI Ruige JB Bouckaert JJ Tudor-Locke CE Kaufman JM De Bourdeaudhuij IM The effects of a pedometer-based behavioural modification program with phone support on physical activity and sedentary baheviour in type 2 diabetes patients Patient Edu 2011 84 Suppl 2 275 279 10.1016/j.pec.2010.07.010 20732776

[B17] John D Thompson DL Raynor H Bielak KM Bassett DRJ Effects of treadmill workstations as a worksite physical activity intervention in overweight and obese office workers J Phys Act Health 2011 8 Suppl 8 1034 1043 22039122 10.1123/jpah.8.8.1034

[B18] Schulz K Altman D Moher D Group TC CONSORT 2010 Statement: updated guidelines for reporting parallel group randomised trials BMC Med 2010 8 18 10.1186/1741-7015-8-18 20334633 PMC2860339

[B19] Marshall AL Miller YD Burton NW Brown WJ Measuring total and domain-specific sitting: a study of reliability and validity Med Sci Sports Exerc 2010 42 1094 1102 19997030 10.1249/MSS.0b013e3181c5ec18

[B20] Atkin AJ Gorely T Clemes SA Yates T Edwardson C Brage S Salmon J Marshall SJ Biddle SJH Methods of measurement in epidemiology: sedentary behaviour Int J Epidem 2012 41 1460 1471 10.1093/ije/dys118 PMC3465769 23045206

[B21] Chau JY Van Der Ploeg HP Dunn S Kurko J Bauman AE Validity of the occupational sitting and physical activity questionnaire Med Sci Sports Exerc 2012 44 Suppl 1 118 125 21659903 10.1249/MSS.0b013e3182251060

[B22] Donovan RJ Jones S Holman CD Corti B Assessing the reliability of a stage of change scale Health Educ Res 1998 13 285 291 10.1093/her/13.2.285 10181026

[B23] Puig-Ribera A Peña O Romaguera M Duran E Heras A Solà M Sarmiento M Cid A How to identify physical inactivity in Primary Care: validation of the Catalan and Spanish versions of 2 short questionnaires Aten Primaria 2012 44 Suppl 8 485 493 22463945 10.1016/j.aprim.2012.01.005 PMC7025196

[B24] Tudor-Locke C Williams JE Reis JP Pluto D Utility of pedometers for assessing physical activity: convergent validity Sports Med 2002 32 Suppl 12 795 808 12238942 10.2165/00007256-200232120-00004

[B25] Kleinbaum DG Kupper LL Morgenstern H Epidemiologic Research 1982 Belmont CA: Lifetime Learning Publications 320 376 343,419–456

[B26] Armitage P Berry G Matthews JNS Statistical Methods in Medical Research 2002 4 Oxford: Blackwell

[B27] Word Medical Association Declaration of Helsinki - Ethical principles for medical research involving human subjects http://www.wma.net/en/30publications/10policies/b3/index.html

[B28] King AC Marcus B Ahn D Dunn AL Rejeski WJ Sallis JF Coday M Activity Counseling Trial Research Group Identifying subgroups that succeed or fail with three levels of physical activity intervention: the activity counseling trial Health Psychol 2006 25 336 347 16719605 10.1037/0278-6133.25.3.336

[B29] Wilcox S Dowda M Dunn A Ory MG Rheaume C King AC Predictors of increased physical activity in the active for life program Prev Chronic Dis 2009 6 A25 19080031 PMC2644611

[B30] Estabrooks PA Glasgow RE Dzewaltowski DA Physical activity promotion through primary care JAMA 2003 289 2913 2916 10.1001/jama.289.22.2913 12799388

[B31] Grandes G Sánchez A Torcal J Ortega R Lizarraga K Serra J The PEPAF Group Targeting physical activity promotion in general practice: characteristics of inactive patients and willingness to change BMC Public Health 2008 8 172 10.1186/1471-2458-8-172 18498623 PMC2412873

[B32] Lewis BS Lynch WD The effect of physician advice on exercise behaviour Prev Med 1993 22 110 121 10.1006/pmed.1993.1008 8475007

